# The effects of 8 weeks of multi-directional movement training combined with balance training on the change of direction of young table tennis players

**DOI:** 10.3389/fphys.2025.1541639

**Published:** 2025-01-28

**Authors:** Qianqian Chen, Yanfei Li, Xinchen Heng, Lei Zhao, Bin Wu

**Affiliations:** ^1^ P.E. Department, Nanjing University of Science and Technology, Nanjing, China; ^2^ Department of Physical Education, Jiangsu Maritime Institute, Nanjing, China; ^3^ Department of Physical Education, Jinling Institute of Technology, Nanjing, China; ^4^ School of Sports Training, Tianjin University of Sport, Tianjing, China; ^5^ Department of Physical Education, Nanjing City Vocational College, Nanjing, China

**Keywords:** change of direction, multi-directional movement training, balance training, young athletes, table tennis, agility

## Abstract

**Background:**

Change of direction (COD) skills are crucial for athletic performance in sports such as table tennis. Improving COD can enhance overall agility and responsiveness during competition.

**Objective:**

To investigate the effects of an 8-week multi-directional movement training combined with balance training on the COD performance of young table tennis players.

**Methods:**

Thirty young table tennis players from the same team were randomly assigned to two groups: the multi-directional movement training combined with the balance training group (MB, n = 15) and the control group (CON, n = 15). The MB group underwent balance training on unstable surfaces, while the CON group performed the same balance exercises on stable ground. Both groups participated in the same multi-directional movement training program, which was conducted three times a week with 24–48 h of recovery between sessions for a duration of 8 weeks.

**Results:**

Significant group effects were observed in the modified agility test, non-dominant leg, dominant leg, and push block side lunge right test (p < 0.05). No significant group effects were found for the hexagon agility test, 3 m side slide test, A-movement test, or the whole table variable speed pendulum test (p > 0.05). Significant time-by-group interactions were noted for all variables (p < 0.05), indicating that improvements over time differed between the MB and CON groups. In the MB group, significant improvements were observed across all tests post-intervention, with high effect sizes (Partial η^2^ values ranging from 0.361 to 0.815). In contrast, the CON group showed significant time effects in only a few tests, including the 3 m side slide test, A-movement test, modified agility test, and push block side lunge right test (p < 0.05), with no significant time effects for other variables.

**Conclusion:**

This study aimed to explore whether multi-directional movement training, when combined with balance exercises, could improve COD skills in young table tennis players. The results will inform future training strategies for enhancing agility and movement efficiency in table tennis athletes.

## 1 Introduction

Table tennis is a high-intensity, fast-paced sport that demands exceptional agility, balance, and change of direction (COD) abilities (Ozmen and Aydogmus, 2016). The game’s dynamic nature demands rapid movements in multiple directions, quick reactions, and precise body control (Huang, 2022). Developing effective training strategies to enhance these physical attributes is crucial for improving performance and reducing injury risk in young table tennis players. Change of direction ability, in particular, is a fundamental skill in table tennis that allows players to quickly adjust their position and respond to opponents’ shots (Dos’ Santos et al., 2019). This skill involves a complex interaction of various physical components, including strength, power, balance, and coordination (Young and Rogers, 2014). While traditional training methods often focus on isolated aspects of physical fitness, such as strength or endurance, recent research suggests that an integrated approach, combining multiple physical components, may be more effective in improving sport-specific performance (Aloui et al., 2021).

Multi-directional movement training has gained attention recently as a potential method for enhancing COD ability in various sports (Jlid et al., 2020). For instance, short-term plyometric jump training has been shown to significantly improve physical fitness components such as strength, power, agility and repeated-sprint ability in young soccer players (Negra et al., 2020; Negra et al., 2017), These findings underscore the potential of targeted plyometric exercises to improve sport-specific performance in youth populations. This type of training involves exercises that challenge athletes to move quickly and efficiently in multiple planes of motion, mimicking the demands of their sport (Elgammal and Radwan, 2022). By incorporating multi-directional movements into training programs, athletes may develop improved neuromuscular control, proprioception, and overall movement efficiency (Guo et al., 2021). Balance training, on the other hand, has long been recognized as an essential component of athletic development and injury prevention programs. Improved balance can contribute to better postural control, stability during dynamic movements, and overall performance in sports that require quick changes in direction (Giboin et al., 2015). In table tennis, where players must maintain stability while executing fast and precise movements, balance training plays a crucial role in supporting the execution of high-intensity actions. In table tennis, where players must maintain stability while executing fast and precise movements, balance training is particularly important in supporting high-intensity actions.

The combination of multi-directional movement training and balance training has demonstrated promising results in improving athletic performance across various sports. For instance, Aloui et al. (2022) found that the integration of plyometric and short sprint training significantly enhanced measures of jump height, speed, COD, and balance in young soccer players. Similarly, Vuong et al. (2023) reported that combined change of direction and plyometric training on sand enhanced jumping, sprinting, and COD performance in professional basketball players. Combined balance and plyometric training has been shown to improve athletic performance metrics such as agility, jumping ability, and stability in female basketball players (Bouteraa et al., 2020). However, despite the growing body of evidence supporting the efficacy of multi-directional movement and balance training in other sports, there is a notable lack of research specifically addressing the needs of young table tennis players. Table tennis presents unique challenges due to its fast-paced nature and the need for precise movements within a confined space (Kaabi et al., 2022). As such, it is crucial to investigate whether the benefits observed in other sports can be translated to the specific demands of table tennis.

Given the potential benefits of combined multi-directional movement and balance training, and the absence of research targeting young table tennis players, there is a clear need for further investigation in this area. Understanding the effects of such a training program on COD ability in young table tennis players could provide valuable insights for coaches and practitioners seeking to optimize performance in this population. The present study aims to assess the effects of an 8-week multi-directional movement training program, coupled with balance training, on COD ability in young table tennis players. By focusing on this specific population and tailoring the intervention to the unique demands of table tennis, this study seeks to provide evidence-based recommendations for enhancing performance in this fast-paced, dynamic sport. It is hypothesized that 8 weeks multi-directional movement training combined with balance training can improve the COD performance of young table tennis players significantly.

## 2 Materials and methods

### 2.1 Participants

The study group consisted of 30 young Chinese table tennis players from a club in Zhejiang, born between 2007 and 2010, who were training in table tennis at the specialized sports training stage (Table 1), determined using G-Power (version 3.1.9.7; Franz Faul, University of Kiel, Kiel, Germany). These calculations were based on an α error probability of 0.05, a power (1-β error probability) of 0.8, an effect size (ES) of 0.4, and a test family encompassing F-tests and analysis of variance (ANOVA), specifically focusing on repeated measures and within-between interaction (Beck, 2013). The study group was selected arbitrarily using the following criteria: Written consent from parents and manager to participate in the research, membership in the province team, a minimum 3-year training period, health conditions, allowing all physical fitness tests to be carried out, the dominant arm is right side, have not suffered from any lower limb injury related to balance loss within the past 3 years, and playing style, requiring the use of rackets with a so-called smooth lining (excluding people using rackets with atypical cladding, such as anti-spin cladding, short pin, or long pin, where play is characterized by a different technique than topspin strokes used in a battery of special tests). The study was conducted in compliance with the Declaration of Helsinki and was approved by the local ethics committee (TJUS-2024-050). All data were analyzed confidentially.

**TABLE 1:** Physical characteristics of participants in the MB and CON groups.

	Age (year)	Height (cm)	Mass (kg)	Training experience (years)
MB (n = 15)	15.63 ± 0.88	167.56 ± 2.83	56.75 ± 0.66	3.88 ± 0.66
CON (n = 15)	15.56 ± 0.81	167.44 ± 3.44	57.25 ± 3.34	3.94 ± 0.47

### 2.2 Procedures

**FIGURE 1:**
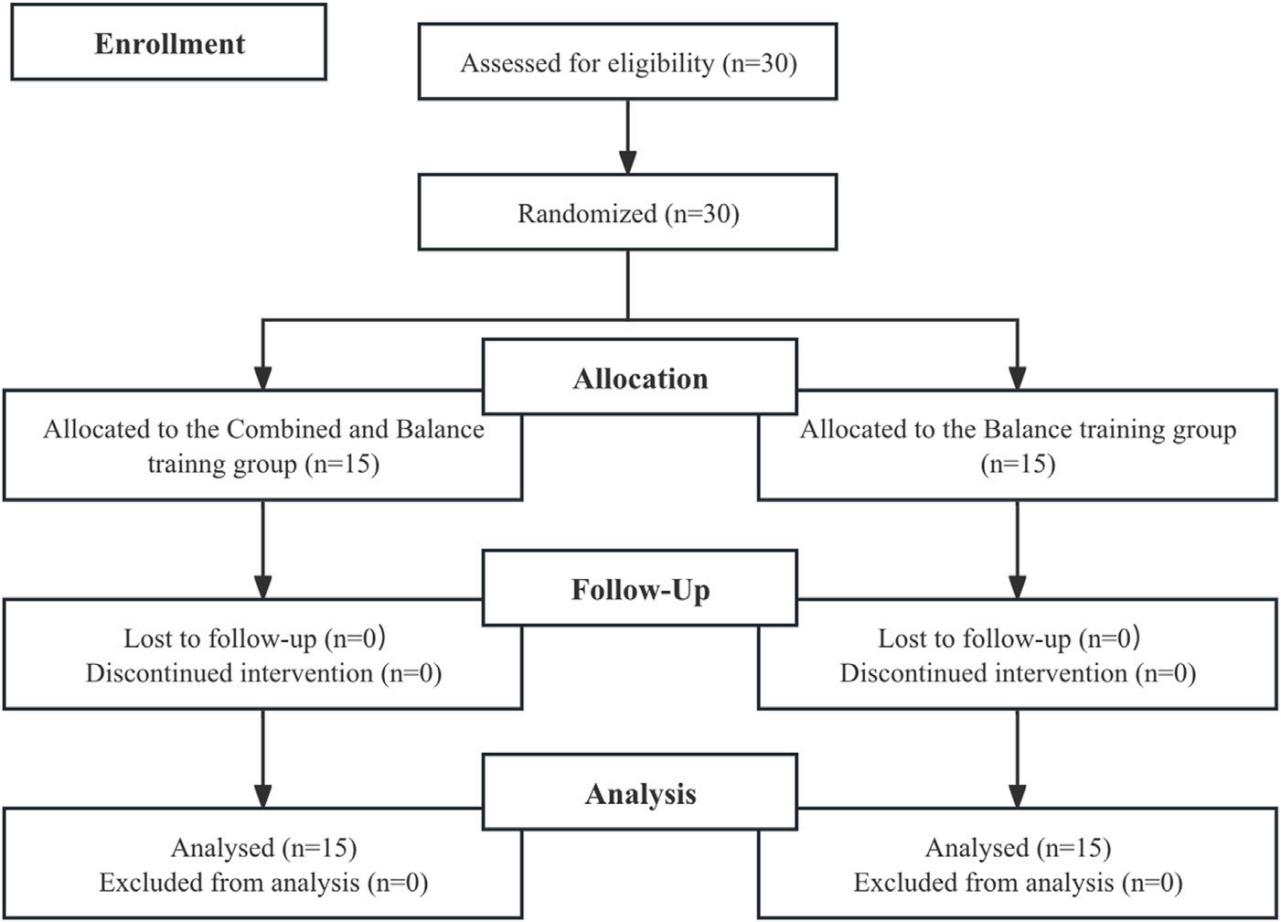
Flow chart of the progress through the phases of the study according to the consort statements.

A total of 30 players volunteered for random allocation into two groups: the multi-directional movement training combined with balance training group (MB, n = 15) and a control group (CON, n = 15) according to a computer-generated randomization list (See Figure 1 for the recruitment process). Both groups performed the training program three times per week, with 24–48 h of recovery between sessions. The MB group engaged in a balance training program (Table 2), which was conducted under unstable conditions, while the CON group performed the same balance training program on a stable surface (floor). Following this, both the MB and CON groups participated in an identical multi-directional movement training program.

**TABLE 2:** The balance training program for multi-directional movement training combined balance training group and multi-directional movement training group.

Exercises	The first stage (1–2 weeks)	The second stage (3–5 weeks)	The third stage (6–8 weeks)
Stand on the balance board exercise	Static standing on the board with two legs 30 s/set*3 set	Static standing on the board with two legs and eyes closed 30 s/set*3 set	Squat on the plate with eyes closed (3 sets: 10 reps/set)
Supine straight leg bridge on Swiss Ball	Isometric supine straight leg bridge on Swiss Ball 30 s/set*3 set	Isometric supine single-leg bending bridge on Swiss Ball 30 s/set*3 set	Dynamic supine single-leg bending bridge on Swiss 10 reps/set*3 set
Side-plank with inflated balance disc	Side-plank with inflated balance disc with elbow 30 s/set*3 set	Side-plank with inflated balance disc and the non-supporting leg stretches backward 30 s/set*3 set	Side-plank with inflated balance disc and the non-supporting leg stretches backward with elastic band 10 reps/set*3 set
Lunge squat on BOSU ball	Lunge squat on BOSU ball 10 reps/leg/set*3 set	Lunge squat on BOSU ball and inflated balance disc 10 reps/leg/set*3 set	Lunge squat on BOSU ball and inflated balance disc with 5 kg dumbbells 10 reps/leg/set*3 set
Airex^®^ Balance-pad Elite exercise	Single-leg squat with balance-pad 10 reps/leg/set*3 set	Single-leg standing with balance-pad and the non-supporting leg stretches backward 12 reps/leg/set*3 set	Single-leg support with balance-pad elite and the non-supporting leg stretches backward with elastic band 12 reps/leg/set*3 set
Rest	Between exercise: 60 s Between sets: 3 min		

Note: MB, group conducted training program on unstable support (e.g., BOSU, ball, Swiss ball, and Balance pad); and CON, group conducted training program on stable support (i.e., solid floor.

Before the official start of the training and testing, all participants underwent a 2-week familiarization phase, which involved training three times per week. During this period, participants were introduced to the balance exercises, multi-directional movement drills, and the test procedures. In addition, they received guidance on proper technique from a certified strength and conditioning coach. All training sessions and testing protocols were supervised by study personnel with expertise in strength and conditioning. Detailed descriptions of the balance training and multi-directional movement training protocols are provided in Tables 2, 3.

**TABLE 3:** The multi-directional movement training (MB) program for MB and CON group.

Exercises	The first stage (1–2 weeks)	The second stage (3–5 weeks)	The third stage (6–8 weeks)
Front barrier jump	Front high leg raise (15 cm) (15 hurdles/set*3 sets)	Front high leg raise (23 cm) (20 hurdles/set*3 sets)	Front high leg raise (30 cm) (25 hurdles/set*3 sets)
Quadrant jumps	Triangle jumps on both feet (4 round*3/set*3 sets)	Quadrilateral jumps on both feet (6 round *3/set*3 sets)	Hexagonal jumps (8 round *3/set*3 sets)
Cross jumps +10 m sprint	(4 round + sprints)/set * 3 sets	(6 round + sprints)/set * 3 sets	(8 round + sprints)/set * 3 sets
“Z” sprint	“Z” sprint + Forward rail jumps (15 cm) (2 reps/set*3 sets)	“Z” sprint + Forward rail jumps (23 cm) (3 reps/set*4 sets)	“Z” sprint + Forward rail jumps (30 cm) (4 reps/set*5 sets)
“M” sprint	“M” sprint + Lateral barrier jumps (15 cm) (2 reps/set*3 sets)	“M” sprint + Lateral barrier jumps (23 cm) (3 reps/set*4 sets)	“M” sprint + Lateral barrier jumps (30 cm) (3 reps/set*4 sets)
Lateral slide folding move + Sprint	(3 m return*4+5 m sprint)/set*3 sets	(3 m return*5+5 m sprint)/set*3 sets	(3 m return*6+5 m sprint)/set*3 sets
one-step lateral stomp	15 reps/set * 3 sets	20 reps/set * 3 sets	25 reps/set * 3 sets
*In-situ* crossover stomp swing	15 reps/set * 3 sets	20 reps/set * 3 sets	25 reps/set * 3 sets
Signal indeterminate movement	2-3 signal responses 20 s/set * 3 sets	4-5 signal responses 20 s/set * 3 sets	5-6 signal responses 20 s/set * 3 sets
Intensity	Low intensity	Middle intensity	High intensity
Rest	Between exercise: 60 s Between sets: 3 min	Between exercise: 60 s Between sets: 3 min	Between exercise: 60 s Between sets: 3 min

#### 2.2.1 Hexagon agility test

**FIGURE 2:**
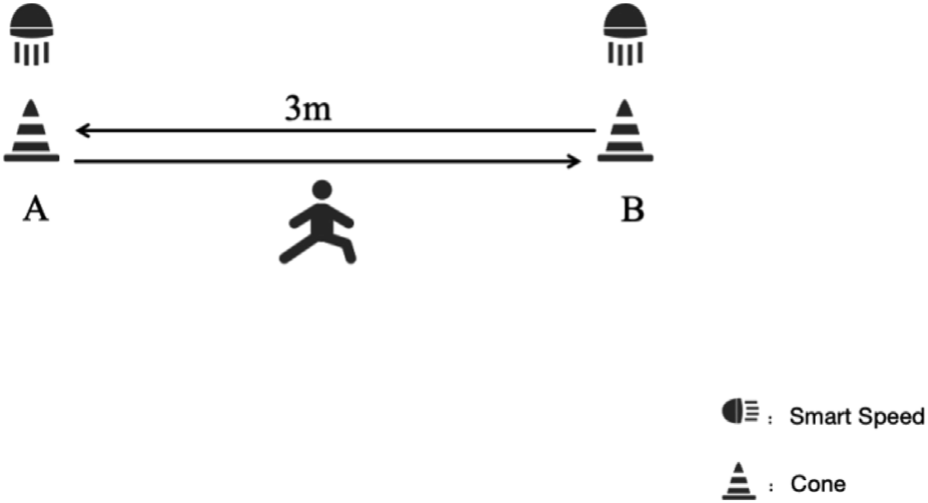
Hexagon agility test design.

The Hexagon Agility Test is a widely used assessment tool for evaluating players’ COD ability and has been validated as an effective method for assessing on-court performance (Beekhuizen et al., 2009). For the test, participants began by standing 50 cm behind the No. 1 side of the hexagon (Figure 2). Upon hearing the command “Ready, go,” they were required to jump in and out of the lines in a clockwise direction, moving from 1 to 6. The test was timed using Smart Speed (Fusion Sport, Coopers Plains, Australia), which automatically recorded the start and end times. Each participant completed the test three times, and the shortest time recorded was used for analysis. A 2-min passive rest period was provided between each trial to ensure sufficient recovery.

#### 2.2.2 T-half change of direction speed test

**FIGURE 3:**
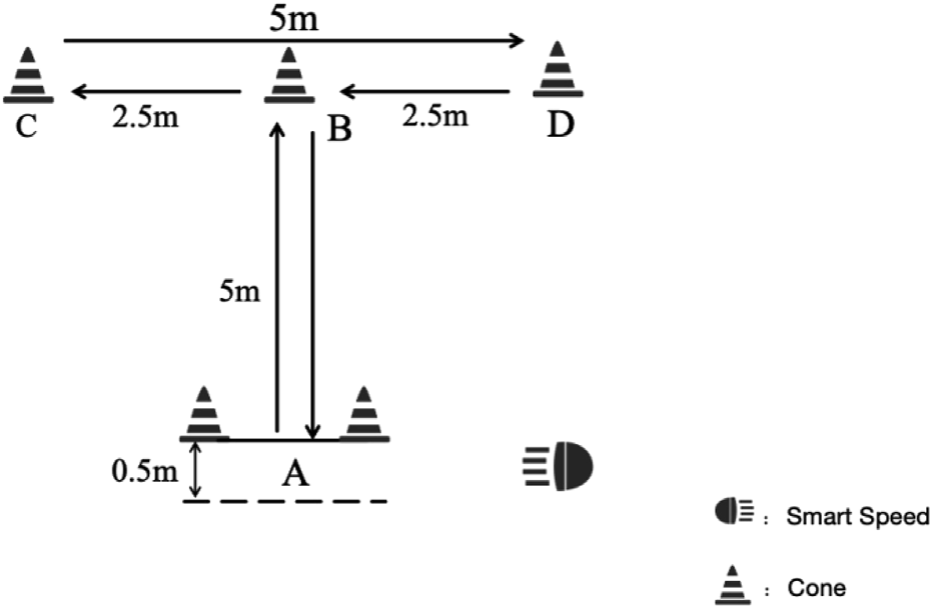
T-half change of direction speed test design.

The T-half change of direction speed test was used to assess table tennis player’s CODA ability (Picabea et al., 2021). Total distance of the test was 20 m. The participants’ movements during the MAT were as follows (Figure 3): 1) A-B movements (5 m): Participants sprinted forward to cone B and touched the top of it with the right hand; 2) B-C movements (2.5 m): Moving laterally without crossing the feet, participants ran to cone C and touched its top with the left hand; 3) C-D movements (5 m): Participants ran laterally to cone D and touched its top with the right hand; 4) D-B movements (2.5 m): Participants moved back to cone B and touched its top with the left hand; 5) B-A movements (5 m): Participants ran backwards to line A. Trials where participants crossed their feet during B-C, C-D, and D-B movements, failed to touch the top of the cone, and/or failed to face forward throughout the tasks, were repeated. Each participant performed three trials interspersed with a 2 min rest period. One photocell gate was used to record the Smart Speed (Fusion Sport, Coopers Plains, Australia). The best value was selected for further analysis.

#### 2.2.3 3 m side slide test

**FIGURE 4:**
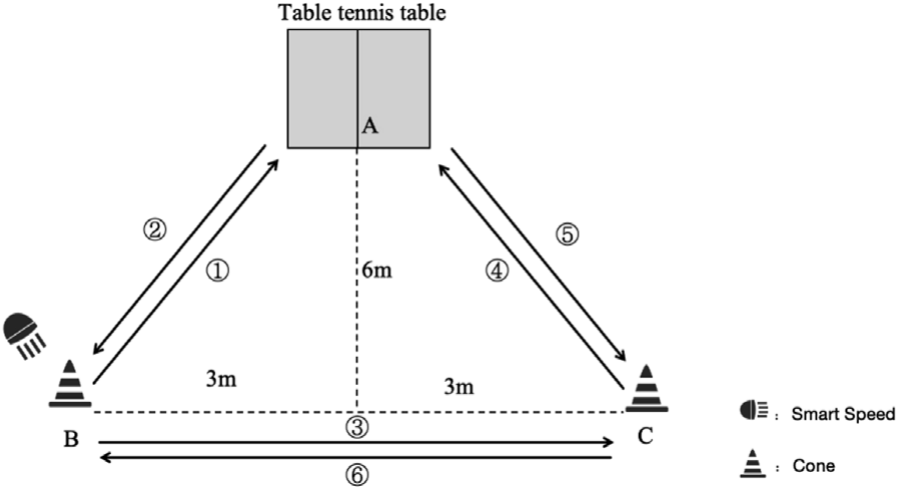
3 m side slide test design.

The 3 m side slide test is used to assess a table tennis player’s change of direction ability. In a table tennis court, two cones (designated A and B) were positioned at a distance of 3 m apart (Figure 4). Smart Speed (Fusion Sport, Coopers Plains, Australia) was placed behind each of the cones. Prior to the commencement of the test, the participant was positioned between the two cones and contacted the upper edge of one side of the cones with their hand. Upon hearing the tester’s “start” command, the subject was required to return to the two cones for five round trips, with each return necessitating contact with the upper edge of the baffle plate for the test to be considered valid. Each subject will complete a total of three maximal effort tests, with the highest score from the three tests being considered the final valid score. A recovery period of five to 10 minutes will be allowed between each test. The duration of the test was automatically recorded by the Smart Speed apparatus at the commencement and conclusion of the session.

#### 2.2.4 A- movement test

**FIGURE 5:**
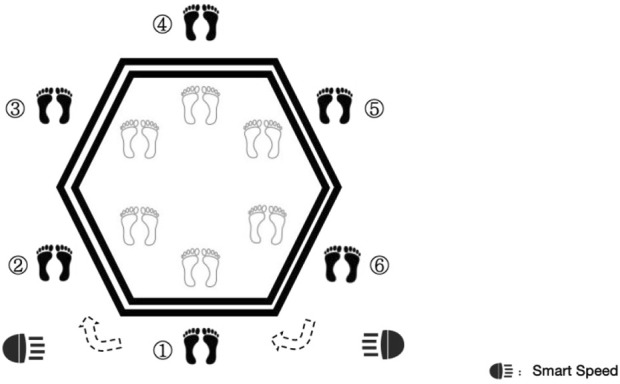
A-movement test design.

The A-movement test is used to assess the ability of table tennis player’s multi-directional change of direction and multi-directional sprint capability (Figure 5). In a table tennis court, the midpoint of the end line of the table tennis table is designated as point A. Perpendicular to the end line, parallel lines are established, extending outward for a distance of 6 m parallel to the end line. Point B is located 3 m on either side of the midpoint of the aforementioned parallel lines, and point C is similarly positioned 3 m from point B. Buckets are placed at points B and C to serve as markers. Prior to the commencement of the test, the athlete is required to assume a position at point B, with their back to the table tennis table. Once the command to commence has been given by the tester, the participants move with haste to the table and touches any position before returning to point B. The athlete then proceeds to move around the barrels to point C and subsequently touches the table before returning to point C. At the conclusion of the test, the time taken to reach point A is recorded. The test is timed automatically by Smart Speed at both the start and the end of the test. The test is timed automatically by Smart Speed (Fusion Sport, Coopers Plains, Australia) at the beginning and end of the test. The entire test is performed around the marker barrels and cannot be touched, and there is no requirement for footwork throughout the test. Each participant performs a total of three Maximum Effort Tests, with the best of the three tests being the final valid score. A 5–10 min breaks between between each test.

#### 2.2.5 Y-balance test

The lower-quarter Y-Balance dynamic test is a dynamic test that requires subjects to maintain single-leg stance while reaching as far as possible with the contralateral leg in 3 different movement directions (i.e., anterior, posteromedial, posterolateral) (Plisky et al., 2006; Hammami et al., 2016b), the assessment reflects the participant’s lower extremity strength, flexibility, and balance ability (Cook et al., 2010). The Y-Balance balance test was performed using the FMS (Move2Perform, United States) standardized test suite.

Before the test started, participants’ length of the right leg was assessed while in a supine lying position by measuring the distance from the anterior superior iliac spine to the most distal aspect of the medial malleolus. Further, participants practiced six trials per reach direction to get familiarized with the testing procedures. All trials were conducted barefooted. The protocol used for the completion of the YBT is similar to that described previously (Cook et al., 2010). Participants stood on the dominant leg, with the most distal aspect of their great toe on the center of the footplate from the YBT Kit. The participants were then asked to push the reach-indicator block with the free limb in the anterolateral, posteriormedial, and posteriorlateral directions in relation to the stance foot on the central footplate, while maintaining their single-limb stance. A test trial was classified invalid if the participants 1) did not touch the line with the reach foot while maintaining weight bearing on the stance leg, 2) lifted the stance foot from the footplate center, 3) lost balance at any point during the trial, 4) did not maintain start and return positions for one full second, or 5) touched down the reach foot to gain considerable support. The variables of interest for the study included the maximal reach for each direction. The average maximum normalized reach across the three directions was calculated in order to record a composite score for each subject. YBT measures were normalized by dividing each excursion distance by the participant’s leg length, then multiplying by 100. Thus, normalized values can be viewed as a percentage of excursions distance in relation to the participant’s leg length (Makhlouf et al., 2018). The test was demonstrated by a member of the research team prior to the participant completing three practice trials in each direction. Following the completion of the test trials, each participant was given a 1-min rest period and then conducted three test trials in each direction, and the maximum distance achieved in each direction (with a precision of 0.5 cm) was selected for the calculation of the total score. According to previous research (Filipa et al., 2010), a composite score (CS) was calculated and considered as the dependent variable using the following formula:

$$CS=\frac{\;Anterolateral\;Md+Posteromedial\;Md+Posterolateral\;Md}{leg\;length\;*\;3}*100\%$$

Md: maximum distance reached.

### 2.3 Push block side lunge right test

This test is used to measure the change of direction and dynamic balance control of table tennis players. Before the test, the participant stands in a ready-to-strike position on one side of the table tennis table and is served multiple balls by a server located on the opposite side of the table. First, two balls are served to the left side of the participant, and the participant completes a push and a block, and then one ball is served to the right side of the participant, and the participant moves to the right side and strikes the ball quickly. The number of successful shots was recorded by the recorder within 1 min. Each participant performed a total of 3 maximal effort tests, and the best of the 3 tests was taken as the final valid score, with a 5-min recovery period between each test. A 5–10 min breaks between each test.

### 2.4 Whole table variable speed pendulum test

The whole table variable speed pendulum test is used to assess a table tennis player’s ability to observe and anticipate the direction of an opponent’s movement on the court, the trajectory of the ball, the spin of the ball, the direction of the ball, and to rapidly change direction and adjust body position. Before the test commences, the participant will serve the ball with a combination of length and tempo changes. Participant is required to make a rapid judgement based on the trajectory, direction, speed and rotation of the ball, and subsequently adjust body posture and position in order to complete a forehand or backhand attack. The number of successful shots was recorded by the recorder within 1 minute. Each participant was given a total of 3 maximal effort tests, and the best score of the three tests was taken as the final valid score, with a 5-min recovery period between each test. The server for each test was selected at random.

### 2.5 Statistical analysis

Experimental data were processed by IBM SPSS statistical software package (version 26.0, Chicago, IL, United States). All data were presented as “mean ± standard deviation” (M±SD). All data were tested for normal distribution using Shapiro-Wilks test. Outliers, defined as studentized residuals greater than 3 standard deviations from zero, were identified and removed. To examine the effects of the MB on the performance, we firstly performed a two-way repeated-measure ANOVA (group × time). The dependent variable for each model was hexagon agility test, T-half change of direction speed test, 3 m side slide test, a-movement test, dominant leg, non-dominant leg, push block side lunge right test, and the whole table variable speed pendulum test. The model factors were group, time, and their interaction. When a significant interaction was observed, LSD *post hoc* correction was performed to identify the location of the significance. The model factor was time. *Partial η* ^2^ was used to assess the effect size (ES) where the significance was observed, with its strength being interpreted as the following: <0.06 as small, <0.14 as moderate, and ≥0.14 as large (Cohen, 1988). The level of significance was set at *p* < 0.05 for all tests.

## 3 Results

For group effects, significant differences (p < 0.05) were observed in the modified agility test, non-dominant leg, dominant leg, and push block side lunge right test. No significant group effects were found for the hexagon agility test (p = 0.083), 3 m side slide test (p = 0.086), a-movement test (p = 0.084), and the whole table variable speed pendulum test (p = 0.079) (Table 4).

**TABLE 4:** Descriptive statistics of results before and after the 8-week training intervention.

Variable	MB (N = 16)				CON (N = 16)					p-value			
	Pre	Post	Δ	Partial η^2^	Pre		Post	Δ	Partial η^2^	Time		Group	Time* group
Hexagon Agility Test (s)	4.43 ± 1.02	3.43 ± 0.87*	−1.0 ± 0.95	0.361	4.07 ± 0.75		3.87 ± 0.46	−0.2 ± 0.62	0.022	0.001*		0.083	0.027^#^
T-half change of direction speed test (s)	7.02 ± 0.30	6.47 ± 0.29*	−0.55 ± 0.3	0.739	6.98 ± 0.30		6.83 ± 0.31*	−0.15 ± 0.31	0.170	<0.001*		0.235	<0.001^#^
3 m Side Slide Test(s)	8.23 ± 0.41	7.38 ± 0.43*	−0.85 ± 0.42	0.725	8.14 ± 0.67		7.78 ± 0.60*	−0.36 ± 0.64	0.329	<0.001*		0.493	0.001^#^
A- Movement Test(s)	11.26 ± 0.56	10.37 ± 0.56*	−0.89 ± 0.56	0.791	11.16 ± 0.61		10.78 ± 0.51*	−0.38 ± 0.56	0.399	<0.001*		0.506	<0.001^#^
Dominant Leg	97.15 ± 2.33	106.53 ± 4.19*	9.38 ± 3.39	0.657	96.42 ± 3.95		97.31 ± 5.20	0.89 ± 4.62	0.017	<0.001*		0.005	<0.001^#^
Non-Dominant Leg	95.18 ± 2.31	102.04 ± 3.48*	6.86 ± 2.95	0.653	95.31 ± 3.35		95.52 ± 4.29	0.23 ± 3.85	0.002	<0.001*		0.028	<0.001^#^
Push Block Side Lunge Right Test	52.06 ± 3.44	55.50 ± 3.19*	3.44 ± 3.32	0.815	52.25 ± 2.84		54.19 ± 2.46*	1.94 ± 2.66	0.583	<0.001*		0.672	0.001^#^
Whole Table Variable Speed Pendulum Test	58.63 ± 3.28	63.00 ± 3.88*	4.37 ± 3.59	0.411	58.94 ± 3.55		57.69 ± 3.40	−1.25 ± 3.48	0.054	0.028*		0.079	<0.001^#^

* Statistically significant difference between pre-and post-test, *p* < 0.05.

**FIGURE 6:**
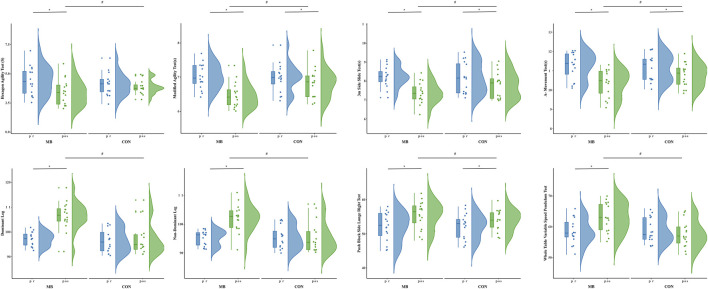
The task performance before and after Training. Note: *p < 0.05.

Significant interactions between time and group were observed across all variables (p < 0.05), indicating that changes over time differed significantly between the MB group and CON group. In the MB, all tests showed significant improvements post-intervention compared to baseline, with the following Partial η^2^ values: hexagon agility test (Partial η^2^ = 0.361), T-half change of direction speed test (Partial η^2^ = 0.739), 3 m side slide test (Partial η^2^ = 0.725), A-Movement Test (Partial η^2^ = 0.791), dominant leg (Partial η^2^ = 0.657), non-dominant leg (Partial η^2^ = 0.653), push block side lunge right test (Partial η^2^ = 0.815), and the whole table variable speed pendulum test (Partial η^2^ = 0.411), all with p < 0.05 (Figure 6).

In the CON, significant time effects (p < 0.05) were observed in the 3 m side slide test (Partial η^2^ = 0.329), a-movement test (Partial η^2^ = 0.399), T-half change of direction speed test (Partial η^2^ = 0.170), and push block side lunge right test (Partial η^2^ = 0.583). However, no significant time effects were found for the hexagon agility test (p = 0.41), dominant leg (p = 0.48), non-dominant leg (p = 0.82), or the whole table variable speed pendulum test (p = 0.20)

## 4 Discussion

The purpose of this study was to investigate the effects of 8 weeks of multi-directional movement training combined with balance training on the COD of young table tennis players. The results of our study demonstrated that the multi-directional movement combined with balance training (MB group) led to significantly greater improvements in COD ability and agility compared to the multi-directional movement-only group (CON group). The MB group showed significant improvements across all performance assessments, including the Hexagon Agility Test, T-half change of direction speed test, and the 3 m Side Slide Test. These findings suggest that integrating balance training into multi-directional movement training can effectively enhance performance in rapid directional changes, which is crucial for table tennis matches.

Due to the small size of the playing table and the high speed at which the game is played, a table tennis player has only a fraction of a second to simultaneously analyze the game, react, move and position properly to hit the ball in optimal conditions (Pradas et al., 2022). All these movement are performed in a limited space and require very fast movement, a high coordinating ability and appropriate strength. Hence the ability to change direction is a key skill required for success of table tennis. This study assessed the change of direction (COD) ability of the subjects during various movements by measuring their performance in the Hexagon Agility Test, T-half change of direction speed test, 3 m Side Slide Test, and A-Movement Test. The results indicate that both training methods effectively improved performance in the T-half change of direction speed test, 3 m Side Slide Test, and A-Movement Test. However, only the MB group showed significant improvement in the Hexagon Agility Test. Furthermore, detailed analysis revealed that the MB group demonstrated significantly greater improvements across all COD test metrics compared to the CON group. This is in line with our expected, and previous research findings in other sports also supported our findings (Bouteraa et al., 2020; Makhlouf et al., 2018).Bouteraa et al. (2020) showed 8-week balance and plyometric training significantly enhanced agility for female adolescent basketball players. Makhlouf et al. (2018) compared that 8-week combination of agility and plyometric training with combination of balance and plyometric training on agility in young soccer players showed that combination of balance and plyometric training was an effective modality to develop agility in young soccer players. Theses research showed the effectiveness of balance training in improve agility. This might be due to the rapid change of direction that occur with agility maneuvers, challenge the ability to either maintain or return the center of gravity over the base of support and thus provide a stress to dynamic balance. Interestingly, this study found that the control group did not show significant improvement in the hexagon agility test, which further highlights the importance of balance in agility-based performance tests. Since the hexagon agility test does not involve long-distance movements and requires quicker footwork, it imposes greater requirements on balance for successful performance.

Additionally, the results of Y-balance test further support our hypothesis. The findings revealed that there was no improvement in the Y-Balance of both dominated and non-dominated leg for the CON group, while the MB group demonstrated significant improvements, and their performance was significantly better than that of the CON group. Zemková and Hamar (2010) demonstrated that after agility-balance training, a significant main effect of time for the dynamic balance in soccer players after 8 weeks of plyometric training on stable and unstable surfaces. Moreover, Bouteraa et al. (2020) also showed that combination of balance and plyometric could significantly improve the Y-balance test performance than regular training. These results were unsurprising, as according to the concept of training specificity (Behm et al., 2015), engaging in balance training is evidently more effective in improving dynamic balance compared to not incorporating balance training. As forementioned, the rapid change of direction movement challenges the ability to either maintain or return the centre of gravity over the base of support and thus provide a stress to dynamic balance. The improvement in dynamic balance observed in this study further supports the enhancement of COD performance. It provides additional evidence that the more effective improvement in COD performance seen in the MB group can be attributed to the enhancement of dynamic balance capabilities.

The results from the Whole Table Variable Speed Pendulum Test and the Push Block Side Lunge Right Test provide valuable insights into the impact of multi-directional movement combined with balance training on table tennis players’ specific performance. The Whole Table Variable Speed Pendulum Test demonstrated a significant improvement in the MB group post-intervention, while the CON group did not show significant improvement. This suggests that the balance training incorporated in the MB group contributed to enhancing the players’ ability to quickly adjust body posture and respond to changes in ball trajectory, speed, and spin. The MB group’s superior performance likely stems from their enhanced neuromuscular control and core stability, developed through the multi-directional movement and balance training protocols. MB have been shown to improve proprioception and dynamic balance, which directly translate to better anticipation and quicker adjustments during gameplay (Hammami et al., 2016a; Bekris et al., 2012).

In the Push Block Side Lunge Right Test, the MB group also showed significant improvements post-intervention, outperforming the CON group, which had less pronounced improvements. The effectiveness of the MB training program is further supported by the specific nature of the exercises, which were designed to mimic the rapid lateral shifts required in table tennis, particularly in blocking and lunging scenarios. Previous studies have demonstrated that multi-directional training improves COD and lateral movements by targeting muscle groups and motor patterns involved in dynamic postural adjustments (Mohr et al., 2024). The program’s focus on balance and lower-body strength likely contributed to the players’ ability to better control their movement and recover more quickly, leading to superior performance in the test (Damian et al., 2024).

There are some limitations in this study. Firstly, although we successfully demonstrated improvements in dynamic balance and agility, it primarily relied on general performance-based tests such as the hexagon agility test and Y-balance test. These assessments, while useful, do not fully reflect the complexity and specificity of table tennis. In table tennis, change of direction involves multiple factors such as ball spin, speed, and placement, which are not adequately captured by the existing tests.Although table tennis-specific tests were incorporated, they were conducted with human-controlled ball serving, making the tests susceptible to variations in the skill level of the server and other human factors. This affects the repeatability and reliability of the tests. Therefore, future studies should focus on developing more precise and standardized table tennis-specific change of direction tests to reduce human interference and provide more reliable measurements. Additionally, another several limitations should be noted.The participant demographics were limited to male athletes from a specific region, which may restrict the generalizability of the findings to other populations, including female athletes or individuals from diverse geographic or athletic backgrounds. The study did not incorporate biomechanical analyses, such as force plate assessments or muscle activation studies, which could have provided a more comprehensive understanding of the underlying mechanisms influenced by the training intervention. The relatively short duration of the study (8 weeks) limits the ability to assess the long-term effects or sustainability of the training outcomes, warranting future longitudinal research. Addressing these limitations in future research could further enhance the understanding and applicability of the findings.

## 5 Conclusion

This study provides compelling evidence that an 8-week multi-directional movement combined with balance training program can significantly improve the COD ability and dynamic balance of young table tennis players. The MB group, which received balance training on unstable surfaces in addition to their regular multi-directional movement training, showed greater improvements across all COD tests compared to the CON group. These results highlight the importance of integrating balance training into table tennis training programs to enhance agility and overall performance.

The findings of this study suggest that multi-directional movement training combined with balance exercises could be an effective method for enhancing the rapid directional changes required in table tennis. These insights provide practical implications for coaches and practitioners seeking to improve performance and reduce injury risk in young athletes.

## Data Availability

The raw data supporting the conclusions of this article will be made available by the authors, without undue reservation.
